# Targeting CB2R in astrocytes for Parkinson's disease therapy: unraveling the Foxg1-mediated neuroprotective mechanism through autophagy-mediated NLRP3 degradation

**DOI:** 10.1186/s12974-023-02989-2

**Published:** 2023-12-19

**Authors:** Hong Zhu, Feng Xiao, Yao Xiao, Yun Guo, Xuesong Shan, Zhe Zhang, Lieliang Zhang, Hua Guo

**Affiliations:** 1 Department of Neurosurgery, The Second Affiliated Hospital, Jiangxi Medical College, Nanchang University, 1# Minde Road, Nanchang, Jiangxi People’s Republic of China; 2 Jiangxi Key Laboratory of Neurological Tumors and Cerebrovascular Diseases, Nanchang, China; 3 Jiangxi Health Commission Key Laboratory of Neurological Medicine, Nanchang, China; 4 Institute of Neuroscience, Nanchang University, Nanchang, China; 5 Department of Anesthesiology, The Second Affiliated Hospital, Jiangxi Medical College, Nanchang University, 1# Minde Road, Nanchang, 330006 Jiangxi People’s Republic of China; 6 Key Laboratory of Anesthesiology of Jiangxi Province, Nanchang, China

**Keywords:** CB2R, NLRP3, Autophagy, Parkinson disease

## Abstract

**Background:**

Inflammasomes in astrocytes have been shown to play a crucial role in the pathogenesis of neurodegenerative diseases such as Parkinson's disease (PD) and Alzheimer's disease (AD). Cannabinoid Receptor 2(CB2R), a G protein-coupled receptor (GPCR), is considered a promising therapeutic target in inflammation-related disorders. This study aims to explore the role of CB2R in regulating NOD-like receptor family pyrin domain containing 3 (NLRP3)-mediated neuroinflammation in astrocytes.

**Methods:**

In an in vivo animal model, specific targeting of astrocytic CB2R was achieved by injecting CB2R-specific adenovirus (or fork head box g1(foxg1) adenovirus) to knock down CB2R or administering CB2R agonists, inhibitors, etc., in the substantia nigra pars compacta (SNc) of mice. A PD mouse model was established using 1-methyl-4-phenyl-1,2,3,6-tetrahydropyridine (MPTP) induction. Animal behavioral tests, western blot, immunofluorescence, and other experiments were performed to assess the loss of midbrain tyrosine hydroxylase (TH) neurons, activation of astrocytes, and activation of the NLRP3 pathway. Primary astrocytes were cultured in vitro, and NLRP3 inflammasomes were activated using 1-methyl-4-phenylpyridinium (MPP^+^) or lipopolysaccharide (LPS) and adenosine triphosphate (ATP). Western blot and ELISA experiments were conducted to assess the release of inflammatory factors. Transcriptomic sequencing and CUT&RUN techniques were employed to study the CB2R regulation of the foxg1 binding site on the autophagy molecule microtubule-associated protein 1 light chain 3 beta (MAP1LC3B).

**Results:**

Astrocytic CB2R knockdown impaired the motor abilities of MPTP-induced mice, exacerbated the loss of TH neurons, and induced activation of the NLRP3/Caspase-1/interleukin 1 (IL-1β) pathway. Activation of CB2R significantly alleviated motor impairments in mice while reducing NLRP3 deposition on astrocytes. In vitro cell experiments showed that CB2R activation attenuated the activation of the NLRP3/Caspase-1/IL-1β pathway induced by LPS + ATP or MPP^+^. Additionally, it inhibited the binding of foxg1 to MAP1LC3B, increased astrocytic autophagy levels, and facilitated NLRP3 degradation through the autophagy–lysosome pathway.

**Conclusion:**

Activation of CB2R on astrocytes effectively mitigates NLRP3-mediated neuroinflammation and ameliorates the disease characteristics of PD in mice. CB2R represents a potential therapeutic target for treating PD.

**Supplementary Information:**

The online version contains supplementary material available at 10.1186/s12974-023-02989-2.

## Introduction

PD is a prevalent age-related movement disorder. Multiple pathological hallmarks of PD, including neuronal loss, formation of neurofibrillary tangles, deposition of Lewy bodies, and abnormal aggregation of α-synuclein, are closely tied to the typical PD symptoms, such as tremors, rigidity, bradykinesia, and balance problems [1, 2]. Neuroinflammation is recognized as a primary pathological feature of PD, involving a complex physiological process in which astrocytes play a pivotal role [3, 4]. Numerous studies have highlighted the potential involvement of astrocyte dysfunction in PD [5, 6]. Despite the intense focus on the involvement of astrocytes in PD in recent research, the precise role of astrocytes in the pathogenesis of PD remains elusive.

Astrocytes, the primary resident support cells in the brain, play crucial roles, including maintaining neural environment stability, providing nutrients to neurons, and participating in immune responses [7, 8]. Faced with "danger signals" such as pathogens and tissue damage, astrocytes adopt an inflammatory polarization phenotype, accompanied by an increase in the production of pro-inflammatory cytokines, such as IL-1β and tumor necrosis factor-α (TNF-α), and enhanced immune responses [9]. Consequently, activated astrocytes, by augmenting their phagocytic capabilities, aid in the clearance of accumulated toxic substances, such as aberrantly aggregated α-synuclein proteins [10, 11]. Mounting evidence strongly supports the notion that both neuroinflammation and astrocyte dysfunction play crucial roles in the pathogenesis of PD. Particularly, the activation of NLRP3 inflammasome in astrocytes has emerged as a key link in this process. The NLRP3 protein is an integral component of the inflammasome, a multi-protein complex responsible for triggering inflammatory responses [12]. Recent studies have shed light on the intricate relationship between NLRP3 and astrocytes in PD [13].

In the context of PD, a critical factor contributing to the progression of neuroinflammation and subsequent neuronal death is the overactivation of the NLRP3 inflammasome-mediated inflammatory pathway in astrocytes. This pathway, triggered by various stimuli such as pathogenic invasion and tissue injury, initiates a multi-step process [14–16]. Upon activation, NLRP3, which reside in the astrocyte cytoplasm, oligomerize to form a multi-protein complex known as the inflammasome. This formation results in the recruitment and activation of pro-caspase-1, which further processes the pro-inflammatory cytokines, IL-1β, and interleukin-18 (IL-18), into their mature and bioactive forms [12, 17]. The subsequent release of these mature cytokines amplifies the inflammatory response, leading to a heightened state of neuroinflammation in PD. Furthermore, the NLRP3 inflammasome can instigate a form of inflammatory cell death, termed pyroptosis, characterized by cell swelling, membrane rupture, and subsequent release of pro-inflammatory cell contents into the extracellular milieu. This exacerbates the inflammatory state and fuels a vicious cycle of ongoing inflammation, neuronal damage, and cell death, contributing to the progressive nature of PD. Thus, the overactivation of the NLRP3 inflammasome in astrocytes has gained recognition as a key player in the neuroinflammatory pathology observed in PD, presenting potential therapeutic targets to mitigate disease progression.

CB2R has emerged as a promising therapeutic target for central nervous system (CNS)-related diseases due to its immunomodulatory and neuroprotective properties [18–20]. CB2R is primarily expressed on immune cells, including microglia and astrocytes, which play critical roles in neuroinflammation and CNS homeostasis [18]. Activation of CB2R has been shown to exert anti-inflammatory effects by suppressing the release of pro-inflammatory cytokines and chemokines from microglia and astrocytes [21, 22]. Interestingly, emerging evidence suggests that CB2R activation can also influence the NLRP3 inflammasome pathway. Studies have demonstrated that CB2R activation could reduce the expression of the NLRP3 protein in glial cells [23]. Activation of the NLRP3 inflammasome leads to the cleavage and secretion of pro-inflammatory cytokines, IL-1β and IL-18, amplifying the inflammatory cascade and causing neuronal damage [24]. By modulating the NLRP3 pathway, CB2R activation may attenuate neuroinflammation and provide neuroprotection in these conditions.

In our research, we have identified the CB2R in astrocytes as a potential target for the treatment of PD. Activation of CB2R led a reduction in the activation of the NLRP3/Caspase-1/IL-1β signaling pathway in the brain. The specific mechanism involves CB2R activation promoting the autophagy–lysosomal pathway degradation of NLRP3. An essential molecule regulating this process is foxg1, which inhibited the transcription of MAP1LC3B by binding to the region between − 1410 bp and − 1650 bp on the MAP1LC3B promoter. This inhibition weakened the autophagic response of astrocytes, consequently blocking the degradation of NLRP3 and triggering neuroinflammation in the brain, exacerbating the pathological symptoms in PD mice. Our investigation elucidated the pathogenic mechanism of PD, thereby offering a scientific basis for clinical therapeutics.

## Materials and methods

### Animals

Three-month-old male C57BL/6J mice were obtained from the Medical Animal Experiment Center of Nanchang University and were bred and maintained in the Animal Resource Centre of the Faculty of Nanchang University. The mice were provided ad libitum access to food and water and housed in a controlled environment with an ambient temperature of 22 °C ± 2 °C, a 12:12-h light/dark cycle, and a humidity of 60%. All animal procedures strictly adhered to the principles outlined in the Guide for the Care and Use of Laboratory Animals (National Institutes of Health, United States), and were conducted in accordance with the guidelines set forth by the Institutional Animal Care and Use Committee of Nanchang University.

### Antibody information

The specifications for the antibodies needed for the experiment are detailed below. This includes the name of each antibody, the supplier from which it was procured, and the respective dilution ratios applied in western blot (WB), immunofluorescence (IF), and immunohistochemistry (IHC) procedures.

**Table:** 

Antibody name	Company	Item number	Application
TH	Millipore	ab152	IHC:1:1000;WB:1:1000
GFAP	Abcam	ab7260	WB:1:1000;IF:1:1000
NLRP1	Proteintech	12,256-1-AP	WB:1:1000
NLRP2	Proteintech	15,182–1-AP	WB:1:1000
NLRP3	AdipoGen	AG-20B-0014-C100	WB:1:1000;IF:1:500
NLRC4	Abcam	ab201792	WB:1:1000
GAPDH	SantaCruz	sc-32233	WB:1:3000
Caspase-1	AdipoGen	AG-20B-0042	WB:1:1000
IL1β	R&D	AF-401-NA	WB:1:1000
MAP1LC3B	Cell Signaling Technology	2775	WB:1:1000;IF:1:500
Foxg1	Invitrogen	PA5-26,794	WB:1:400;;IF:1:200
GFAP	Millipore	MAB360	IF:1:1000

### Adeno-associated virus (AAVs) injection

In order to generate AAVs, mouse small interfering RNA (siRNA) was transfected with packaging vectors (AAV9-GFAP-Null(CB2R)-bGHpolyA/AAV9-GFAP-Null(foxg1)-bGHpolyA). For the microinjection procedure, mice were firstly anesthetized with intraperitoneal pentobarbital sodium and placed in a stereotaxic apparatus. A 1 μL volume of AAVs was injected using a glass electrode, targeting the midbrain at the coordinates: Ap: − 3.1 mm, R(L): 1.3 mm, V: − 4.5 mm. Following the injection, the needle was retained for an additional 2 min. 30 days after the virus microinjection, subacute MPTP mice model was induced.

### Establishment of the subacute MPTP mice model

Four weeks after AAVs injection, the subacute MPTP mouse model was prepared. MPTP at a dose of 20 mg/kg (Selleck, S4732) was subcutaneously injected (i.h.) once daily for 5 consecutive days. Samples were collected 7 days after the last dose.

### Behavioral testing

#### Open field test

This test is commonly used to assess the locomotor activity and exploratory behavior of mice. Mice were placed in an open field chamber (50 cm × 50 cm × 60 cm) and allowed to acclimate for 15 min. The mice were then observed for 5 min while their locomotor activity was recorded and analyzed using openfield software (CleverSys Inc, VA, USA). The total distance traveled and speed of movement were calculated to evaluate the exploratory behavior and motor function of mice.

#### Rotarod test

Prior to the formal experiment, mice underwent a 3-day adaptation and training period. In the formal experiment, mice were placed on a rotarod apparatus and the drum was set to rotate for 3 min, with the speed accelerating uniformly from 5 to 20 rpm over the first 2 min. The system automatically recorded the dwell time on the rotating drum of mice. Each mouse repeated the experiment 3 times, and the average of the three experiments was taken. Mice that did not fall off the drum within 3 min were counted for 180 s.

#### Pole test

The pole test utilized a standardized wooden pole measuring 1 cm in diameter and 50 cm in height to assess the climbing ability of mice. A small wooden ball was positioned at the summit of the pole, serving as the initial starting point for the mice. A custom-made wooden pole with a stable base was placed within a suitable enclosure, and the mouse was gently positioned atop the pole in an upward-facing orientation. During the training phase, mice underwent continuous training over a period of three consecutive days, encompassing two training sessions per day with a 1-min intermission between sessions. Subsequently, in the formal experimental phase, each mouse underwent three separate trials with a 1-min pause between each trial. The total duration required for the mouse to descend from the top of the pole to the ground using all four limbs was meticulously recorded, and the shortest time achieved was subjected to subsequent analysis.

### High-performance liquid chromatography (HPLC)

Striatum tissue samples were subjected to lysis using 0.1 M perchloric acid, with a ratio of 1 mg of sample in 100 μL of perchloric acid. Following centrifugation, the resulting supernatant was analyzed using a water high-performance liquid chromatography-ultraviolet (HPLC–UV) system equipped with a SHI MADZU reversed-phase column (5 μm, 250 × 4.6 mm). The mobile phase consisted of a mixture of 50 mmol/L KH2PO4 (pH 3.2) and acetonitrile in a ratio of 85:15. A flow rate of 1 mL/min was maintained during the analysis. The samples were monitored using a fluorescence detector at a wavelength of 245 nm. The obtained peaks were subsequently quantified.

### Immunohistochemical staining

The extracted whole brain tissue was gradually dehydrated and embedded in OCT glue, and then sliced continuously (25 μm) using a cryostat (Leica CM1950). The collected brain slices were sealed and stored at − 20 °C in a mixture of 0.01 M phosphate-buffered saline (PBS) and glycerol (1:1) until further use. For tissue staining, brain slices were washed three times with PBS, incubated at room temperature with 3% H_2_O_2_ for 15 min, and then sealed with a PBS–Triton solution containing 5% bovine serum albumin (BSA) at room temperature for 1 h. After removing the sealing solution, the corresponding antibodies (glial fibrillary acidic protein, GFAP; TH) were added and incubated overnight at 4 °C. The next day, the slices were washed with PBS and incubated with HRP-conjugated secondary antibodies (1:1000, Invitrogen) at room temperature for 1 h. After washing with PBS three times, the slices were stained in freshly prepared diaminobenzidine substrate buffer in the dark and then mounted. The mounted slices were air-dried in a cool place, gradually dehydrated, and sealed with resin before being observed under a stereomicroscope to evaluate cell morphology and count (Zeiss, Germany).

### Western blotting

RIPA buffer (with protease inhibitor) was added to the cells cultured in a plate, and the cells were lysed on ice for 20 min. The astrocyte were scraped off using a cell scraper and collected in a 1.5 mL EP tube. For tissue protein extraction, RIPA buffer was added to the tissue according to its weight, followed by tissue disruption using an ultrasonic cell disrupter. The protein extract was centrifuged at 16,000*g* for 10 min at 4 ℃, and the supernatant was transferred to another clean 1.5 mL EP tube. A 1 μL sample was taken to measure the protein concentration using the BCA assay. The remaining protein was mixed with 5X loading buffer and boiled at 95 °C for 5 min, and then stored at − 20 °C. Approximately 40 μg of protein was separated by SDS-PAGE using a pre-set program of 80 V for 40 min and 120 V for approximately 60 min. The separated proteins were transferred onto a PVDF membrane (Millipore, USA) using a wet transfer system with a constant current of 200 mA. The whole PVDF membrane was then immersed in a TBST solution (pH 7.4, 10 mM Tris–HCl, 150 mM NaCl, 0.1% Tween-20) containing 5% skim milk for 1 h. After removal from the skim milk, the membrane was washed with TBST to remove the milk, and then incubated overnight at 4 °C with primary antibodies (TH, GFAP, NLRP1, NLRP2, NLRP3, NLRC4, GAPDH, Caspase-1, IL1β, MAP1LC3B, foxg1). The following day, after removal of the primary antibody, the membrane was washed with TBST for 30 min (with a change of wash buffer every 10 min), and then incubated with secondary antibodies at room temperature for 1 h. Finally, the membrane was washed with TBST for 30 min (with a change of wash buffer every 10 min), and then visualized using an ECL substrate. The protein bands were captured using an imaging system, and the Image J software was used for image analysis. The grayscale values of the target protein were semi-quantitatively analyzed by normalizing to the grayscale values of the internal control GAPDH.

### Immunofluorescence staining of brain tissue sections

The preparation of brain tissue sections is the same as described above for immunohistochemistry. Brain sections were washed three times with PBS, blocked with phosphate buffer solution-Tween-20 (PBST) containing 5% bovine serum albumin (BSA) at room temperature for 1 h, and then incubated with the appropriate primary antibodies (GFAP, NLRP3, Foxg1, MAP1LC3B) overnight at 4 °C. After removing the primary antibodies, the sections were incubated with fluorescent secondary antibodies (Invitrogen, 1:1000) at room temperature for 1 h in the dark. After removing the secondary antibodies, the sections were washed three times with PBS and then stained with Hoechst (Sigma, B2261) to label cell nuclei. Finally, the sections were mounted on slides and examined using confocal microscopy (Zeiss, Germany) with the use of an anti-fluorescence quenching reagent.

### Real-time fluorescent quantitative PCR (real-time PCR)

Total RNA was extracted from midbrain tissues and astrocyte using Trizol Reagent, followed by reverse transcription into cDNA (complementary DNA) using PrimeScript™ RT Master Mix. Real-time qPCR analysis was performed on a StepOnePlus instrument with SYBR Green Master Mix (Applied Biosystems). The com parative 2^(− ΔΔCt)method was employed to determine relative gene expression, utilizing glyceraldehyde-3-phosphate dehydrogenase (GAPDH) as a normalization control. Primers, sourced from Generay (Shanghai, China), were purchased, validated, and utilized with the following sequences:

**Table:** 

Gene name	Forward (5′-3′)	Reversed (5′-3′)
Nog	ACACAAGGGACAAGAGGAATCC	GGGTCAGGTCTTTGCTTCCA
Diras2	CACATGGAAGTGTGCCTTCAT	GATCTGGAGACTCACGGTCCT
Nqo1	GAAGAGCACTGATCGTACTGGC	GGATACTGAAAGTTCGCAGGG
Bambi	TCTGAGCTTAGTGCCTGCTTC	ACGTCATGCAGTCCTCGATAA
Foxg1	CACTTTGAGTTACAACGGGACC	CGAGTTTTGAGTCAACACGGA
Dmpk	CTGCTCGACCTTCTCCTGG	CACGCCCGATCACCTTCAA
Tagln	GCTATGGCATTAACACCACGG	CCCAGGTTCATTAGTGTCCGC
Acta2	CCCAGACATCAGGGAGTAATGG	TCTATCGGATACTTCAGCGTCA
Pde2a	GCCGTTATCGACATTGCTGG	CCCCATCTAGCAGGTAGGTGTA
Ifnb1	CTCACCTACAGGGCGGACT	GGCAAAGGCAGTGTAACTCTT
Fos	AACAGATCCGAGCAGCTTCTA	TTTTGAGCTTCAACCGGCATC
Itgb2	CACTGTCTCAGTTGTGTACCAAG	GCTCTGGTGTATCACAGCGAA
Lacc1	TTGAATGCTGTCCAATACCACC	CCATCCCTTTCATAGCTGATGTT
Cybb	AGTGCGTGTTGCTCGACAA	GCGGTGTGCAGTGCTATCAT
Ctss	CCATTGGGATCTCTGGAAGAAAA	TCATGCCCACTTGGTAGGTAT
Lyn	AACTGCTTCAAACCCATGCG	GGACACAGGACTTCCTCACC
Pik3ap1	GTCCCGGATGCCTCTTTCTC	CACAAGTCATTTCCTGCCAGT
Acod1	GGCACAGAAGTGTTCCATAAAGT	GAGGCAGGGCTTCCGATAG
Tyrobp	CCCAAGATGCGACTGTTCTTC	GTCCCTTGACCTCGGGAGA

### Enzyme-linked immunosorbent assay (ELISA)

The midbrain tissue of experimental mice was extracted, and the protein content was unified by BCA protein quantification experiment, the concentration of IL-1β (R&D, MHSLB00), TNF-α (R&D, DY410), IL-6 (R&D, M6000B)were measured by mouse IL-1β, TNF-α, IL-6 ELISA Kits according to the manufacturer's instructions.

### The primary culture of astrocytes

Newborn mice aged 1–3 days were subjected to gentle meningeal dissection using ophthalmic forceps, scissors, and other tools. Subsequently, the brain tissue was carefully peeled off and placed in a 0.25% trypsin solution (Gibco, 15050065). The tissue was then incubated in a cell culture incubator at 37 °C for 5 min. After the incubation period, the trypsin reaction was stopped by adding complete culture medium, and the cell suspension was filtered through a cell strainer (Biosharp). The filtrate was collected and subjected to centrifugation at 1000*g* for 10 min. The supernatant was discarded, and the cells were resuspended in complete culture medium. The cells were then seeded in T-75 cell culture flasks containing 10% fetal bovine serum (FBS) (Sigma, F8318)-F12/DMEM culture medium and were subsequently maintained through medium changes and passaging for experimental use.

### Cell supernatant extraction

One mL of the collected cell supernatant was subjected to centrifugation at 13,000*g* for 5 min, and 600 µL of the resulting supernatant was cautiously transferred to a separate EP tube. To this, an equal volume of pre-chilled methanol and one-fourth volume of chloroform were added, followed by quick vortexing and shaking. The supernatant was subsequently centrifuged at 13,000 rpm for 5 min, and the supernatant carefully discarded. The pellet was resuspended in 500 µL of pre-cooled methanol, the EP tube content was rapidly shaken, and centrifuged at 13,000 rpm for 5 min, and the supernatant again carefully discarded. The centrifuge tubes containing the cell pellet were placed in a 37 ℃ oven for 5 min, followed by the addition of 40 µL of 2.5X loading buffer to dissolve the protein. After boiling at 95 ℃ for 5 min, the precipitate was utilized for immunoblotting.

### Immunofluorescent staining of cells

Primary astrocytes were seeded in 24-well plates, and after treatment, the cell supernatant was removed and the cells were gently washed with PBS three times. Subsequently, the cells were fixed with 4% paraformaldehyde for 20 min, and after removing the paraformaldehyde, the cells were washed with PBS three times. The cells were then blocked with 5% PBST (0.3% Triton PBS) solution for 1 h, followed by the addition of antibodies (NLRP3, MAP1LC3B, Foxg1, GFAP), and incubated at 4 °C overnight. The next day, after removing the primary antibody, the cells were gently washed with PBS three times, and the corresponding fluorescent secondary antibody (Invitrogen, 1:1000) was added and incubated at room temperature for 1 h. The cells were then washed with PBS three times in the dark, and Hoechst was used to label the astrocyte nuclei for 15 min. After washing the cells with PBS three times in the dark, the slides were mounted with anti-fluorescence quenching agent and observed under a confocal microscope (Zeiss, Germany).

### RNA-transcriptomics sequencing analysis

After extracting mRNA from the processed primary astrocytes, the subsequent step involves RNA Library Preparation. In this process, the extracted RNA is converted into a cDNA library. This conversion is achieved through reverse transcription, where RNA is enzy mathematically converted into cDNA using reverse transcriptase enzymes. Subsequently, the cDNA library is fragmented, and adapters are ligated to the fragments, enabling them to be sequenced later on. Next, the cDNA library undergoes high-throughput sequencing technologies. This sequencing generates millions of short reads, each representing fragments of the original RNA molecules present in the sample. Finally, the generated sequencing data are subjected to data processing and analysis using bioinformatics tools and pipelines (R- language analysis) (Detected by Lianchuan Biotechnology). The primary objective is to determine the expression levels of genes and identify differentially expressed genes between samples.

### Cell transfection

siRNA targeting foxg1 siRNA (HanBio, Shanghai, China) was transfected in mouse primary astrocytes using Lipofectamine RNAiMAX (Invitrogen, 13778030) in OPTIMEM reduced serum medium (Gibco) according to the manufacturer’s instructions. siRNA duplexes used were as follows:

**Table:** 

Foxg1 (sense)	GGACAUGGGAGAUAGGAAATT
Foxg1 (antisense)	UUUCCUAUCUCCCAUGUCCTT

Eight hours later, the transfection mixture was removed and cells were further incubated with normal medium for additional 48 h before cell treatment.

### Tandem fluorescent-mRFP-GFP-MAP1LC3B-adenovirus transduction of astrocyte

The primary astrocytes were transfected with a tandem fluorescent mRFP-GFP-MAP1LC3B adenovirus (HanBio, HB-AP2100001) in accordance with the manufacturer’s instructions. The expression of GFP green fluorescence and RFP red fluorescence of cells was indicative of the efficacy and proficiency of the autophagic process. Following the cellular treatment, the specimens were fixed in 4% polymethanol, washed with PBS, stained with Hoechst, and subsequently subjected to microscopic examination. Green fluorescence intensity was quantified to evaluate the degree of autophagy induction.

### CUT&RUN

According to the manufacturer's product instructions (Vazyme, HD101), firstly, pre-processed primary astrocytes (approximately 1*10^6 cells) were collected. Subsequently, the cells were transferred to EP tubes containing pre-processed ConA Beads Pro, gently mixed, and incubated at room temperature for 10 min with gentle shaking 2–3 times during the incubation. After brief centrifugation, the supernatant was discarded. Next, 100 μL of pre-chilled Antibody Buffer was added to each sample, and the cell–bead complex was resuspended. Antibodies were added to the tubes following the recommended immunodensity from the antibody instructions and mixed gently. After another brief centrifugation to collect the liquid at the bottom, the tubes were either left at 2–8 ℃ overnight or incubated at room temperature for 2 h. Then, 1 μL of pG-MNase Enzyme was taken and added to 100 μL of MNase Dilution Buffer. The mixture was further diluted by taking 1 μL and adding it to 100 μL of Dig-wash Buffer. After inverting the tube to mix, it was placed on ice. Subsequently, 100 μL of the pG-MNase Enzyme pre-mixture was added to the subsequent reaction liquid, and the tube was inverted several times to ensure thorough mixing with the cell-bead complex. The incubation was continued at 4 ℃ for 1 h. In the following experimental steps, 1 μL of CaCl_2_ pre-mixture was added and the tube was inverted several times to fragment the cell chromatin. Finally, 100 μL of Stop Buffer was added to extract the DNA fragments from the cells for subsequent experimental detection. Lastly, qPCR quantification was carried out using specific primer pairs and Spike-in DNA primers to obtain △Ct values for further analysis.

### Transmission electron microscopy observation of autophagosomes in mouse midbrain tissue

Mice were anesthetized with pentobarbital sodium and perfused with 2.5% glutaraldehyde solution for tissue fixation. The midbrain tissue, specifically the SNc region, was dissected using a sharp blade to obtain 1 mm^3 tissue blocks, which were then placed in 2.5% glutaraldehyde solution and fixed overnight at 4 °C. Subsequently, the samples were further fixed in 1% osmium tetroxide solution, followed by en bloc staining with uranyl acetate. After dehydration, the samples were embedded in epoxy resin for preservation. Ultrathin sections were cut using an ultramicrotome and stained with lead citrate. The sections were then observed using a transmission electron microscope (JEM-1010, Tokyo, Japan) to visualize the autophagosomes within the tissue.

## Results

### Knockdown of CB2R on astrocytes exacerbates the pathology of MPTP-induced PD mice

**Fig. 1:**
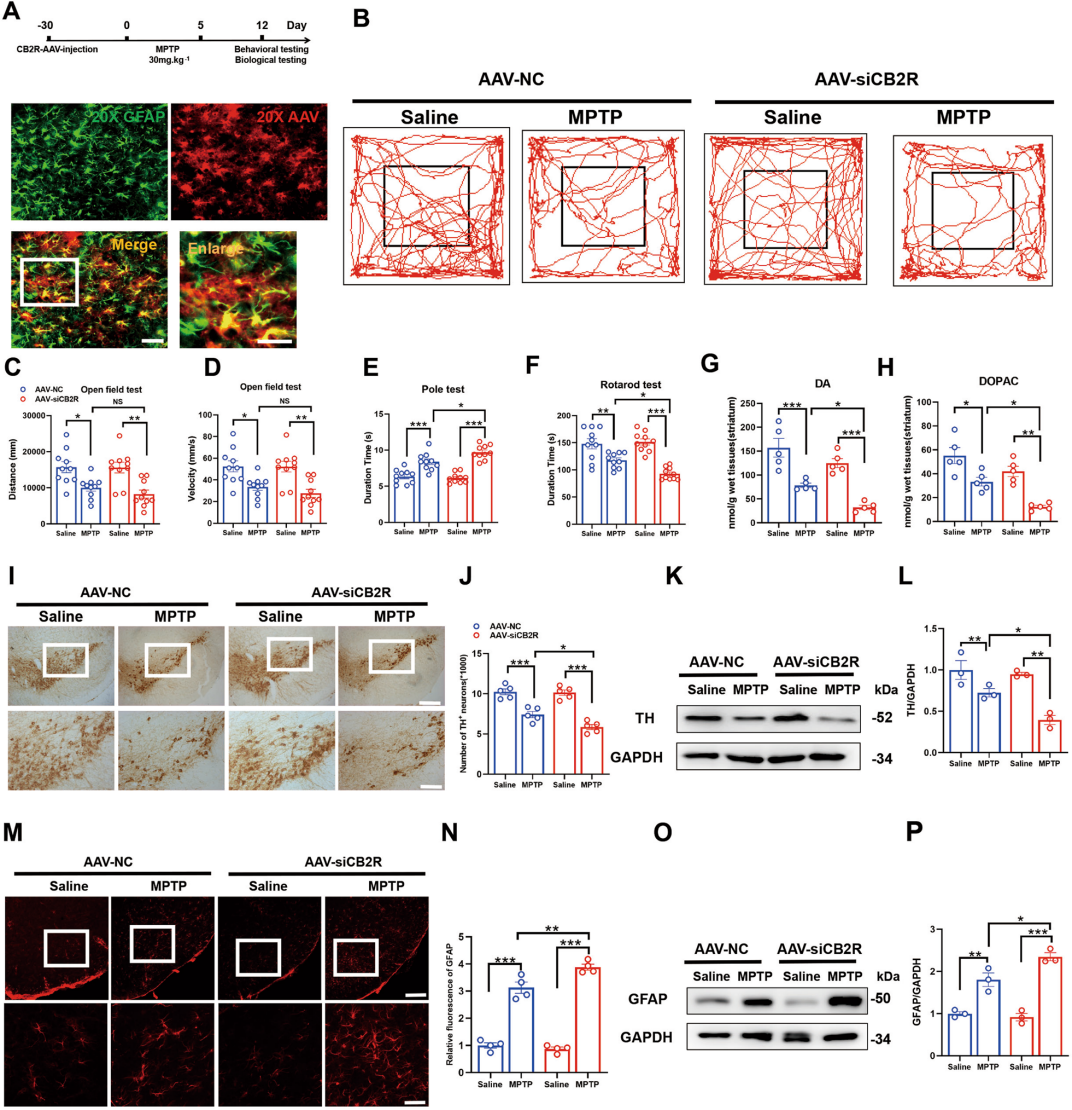
Knockdown of CB2R on astrocytes exacerbates the pathology of MPTP-induced PD mice. **A** Schematic representation of the experimental procedure. The CB2R -AAVs were injected into the midbrain of mice, and after 4 weeks, the mice were induced with MPTP to establish a subacute PD model. Behavioral and biological tests were performed in the following weeks. **B** The trajectory of the mice in the open field test, with statistical data for distance traveled and velocity shown in **C** and **D**, respectively (*n* = 10). Time recorded for each mouse in the pole test (**E**) and rotarod test (**F**), respectively (*n* = 10). **G**, **H** Determination of DA and DOPAC neurotransmitter levels in the striatum of mice by HPLC (*n* = 5). **I** Immunohistochemistry staining for TH-positive neurons in the midbrain of mice (scale bar, 200 μm and 100 μm), with stereological counts shown in **J** (*n* = 5). **K** Detection of TH protein expression in the midbrain of mice by western blot, with quantification shown in **L** (*n* = 3). **M** Immunofluorescence detection of activated astrocytes in the midbrain of mice using GFAP as a marker (scale bar, 200 μm and 100 μm), with fluorescence quantification shown in **N** (*n* = 4). **O** Detection of GFAP protein expression in the midbrain of mice by Western blot, with quantification shown in **P** (*n* = 3). NS means not significant, ^*^P < 0.05, ^**^P < 0.01, ^***^P < 0.001 compared with the corresponding group, as determined by the two-way ANOVA followed by Tukey’s post hoc test

Previous studies have indicated that CB2R on astrocytes play a crucial role in regulating inflammation and their functional properties [21, 25]. Based on these findings, we sought to investigate whether CB2R on astrocytes could influence the pathogenesis of PD. To address this, we employed a viral-mediated knockdown strategy targeting CB2R specifically in astrocytes in mice. Subsequently, the mice were induced to develop a subacute model of PD using MPTP administration (Fig. 1A), and behavioral analyses were performed. Although the targeted knockdown of CB2R did not affect the locomotor activity or speed of the mice in the open field test (Fig. 1B–D), it significantly reduced the time spent in the pole test (Fig. 1E), and more time on rotarod test (Fig. 1F), indicating impaired motor function. Additionally, dopamine (DA), as well as the metabolite 3,4-dihydroxyphenylacetic acid (DOPAC), in the striatal tissue of the mice were measured to gain insights into the underlying PD pathology. The results demonstrated a reduction in striatal dopamine levels following CB2R knockdown (Fig. 1G, H). Furthermore, specific knockdown of CB2R on astrocytes exacerbated the loss of TH neurons induced by MPTP in the SNc (Fig. 1I–L), a region rich in dopaminergic neurons. Immunohistochemical analysis of astrocytes in this region confirmed a more pronounced activation of astrocytes following CB2R downregulation under MPTP-induced conditions (Fig. 1M–P). Collectively, these findings suggested that knockdown of CB2R on astrocytes exacerbated MPTP-induced PD pathology in mice.

### Activation of CB2R as a key mechanism for alleviating neurodegeneration in MPTP-induced PD mice

**Fig. 2:**
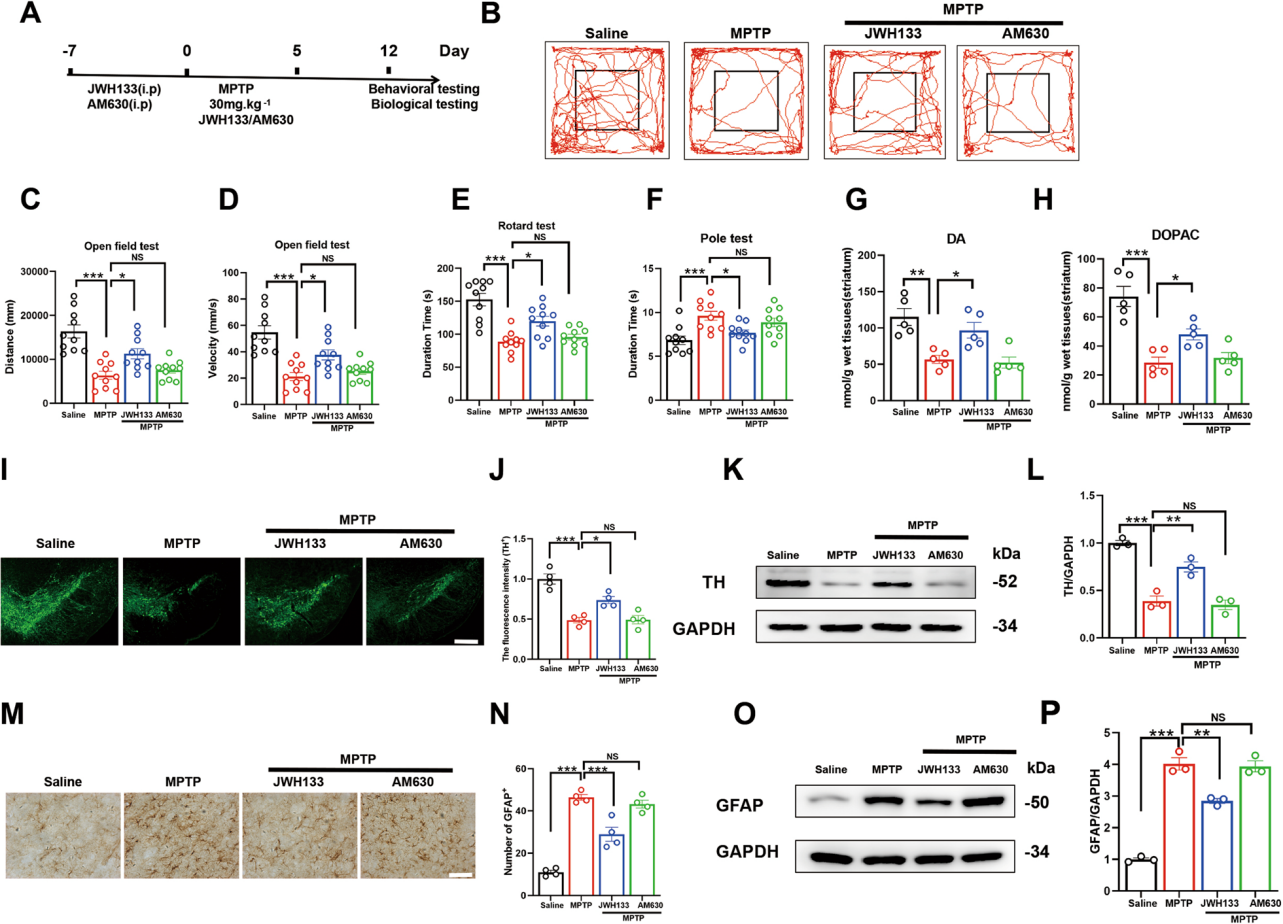
Activation of CB2R as a key mechanism for alleviating neurodegeneration in MPTP-induced PD mice. **A** Subcutaneous injection of MPTP to establish a subacute PD model. During MPTP administration, the relevant groups were also given JWH133 (3 μg) or AM630 (3 μg). Behavioral and biological tests were performed in the following weeks. **B** The trajectory of the mice in the open field test, with distance traveled and time shown in **C** and **D**, respectively (*n* = 10). Time recorded for each mouse in the rotarod test (**E**) and pole test (**F**), respectively (*n* = 10). **G**, **H** Determination of DA and DOPAC neurotransmitter levels in the striatum of mice by HPLC (*n* = 5). **I** Immunofluorescence staining for TH-positive neurons in the midbrain of mice (scale bar, 200 μm), with statistics of data shown in **J** (*n* = 4). **K** Detection of TH protein expression in the midbrain of mice by Western blot, with quantification shown in **L** (*n* = 3). **M** Immunohistochemistry staining for activated astrocytes in the midbrain of mice (scale bar, 200 μm and 100 μm), with quantification shown in **N** (*n* = 4). **O** Detection of GFAP protein expression in the midbrain of mice by western blot, with quantification shown in **P** (*n* = 3). NS means not significant, ^*^P < 0.05, ^**^P < 0.01, ^***^P < 0.001 compared with the corresponding group, as determined by the one-way ANOVA

The effects of activating CB2R vary in different cell types and organisms [26–28]. In neuro-related studies, activation of CB2R has been implicated as an important mechanism for mitigating neuronal damage. To elucidate the functional role of CB2R in the MPTP-induced subacute mice model, mice were administered the CB2R agonist JWH133 and the antagonist AM630 (Fig. 2A), and the associated Parkinson-like pathology was observed. Pre-administration of the CB2R agonist JWH133 was found to improve motor impairments induced by MPTP in mice, as evidenced by increased locomotor activity and speed in the open field test (Fig. 2B–D). Additionally, the time spent on the rotarod increased (Fig. 2E), while the climbing time decreased in pole test (Fig. 2F). However, mice treated with the CB2R antagonist did not exhibit the aforementioned improvements. Consistent with previous findings, HPLC analysis of the striatal tissue revealed a significant increase in dopamine, DOPAC levels following CB2R activation compared to the AAV-NC MPTP group (Fig. 2G, H). Immunofluorescence staining TH neurons in the SNc and western blotting results further demonstrated that CB2R activation alleviated the loss of TH neurons in the midbrain (Fig. 2I–L) and reduced the activation of astrocytes in this region (Fig. 2M–P). Therefore, our results suggested that CB2R activation mitigated Parkinson-like behavioral and pathological symptoms in the MPTP-induced mice, whereas inhibition of the CB2 receptor does not produce similar effects.

### CB2R activation mitigates neuroinflammation in PD through inhibition of the NLRP3/Caspase-1/IL-1β pathway

**Fig. 3:**
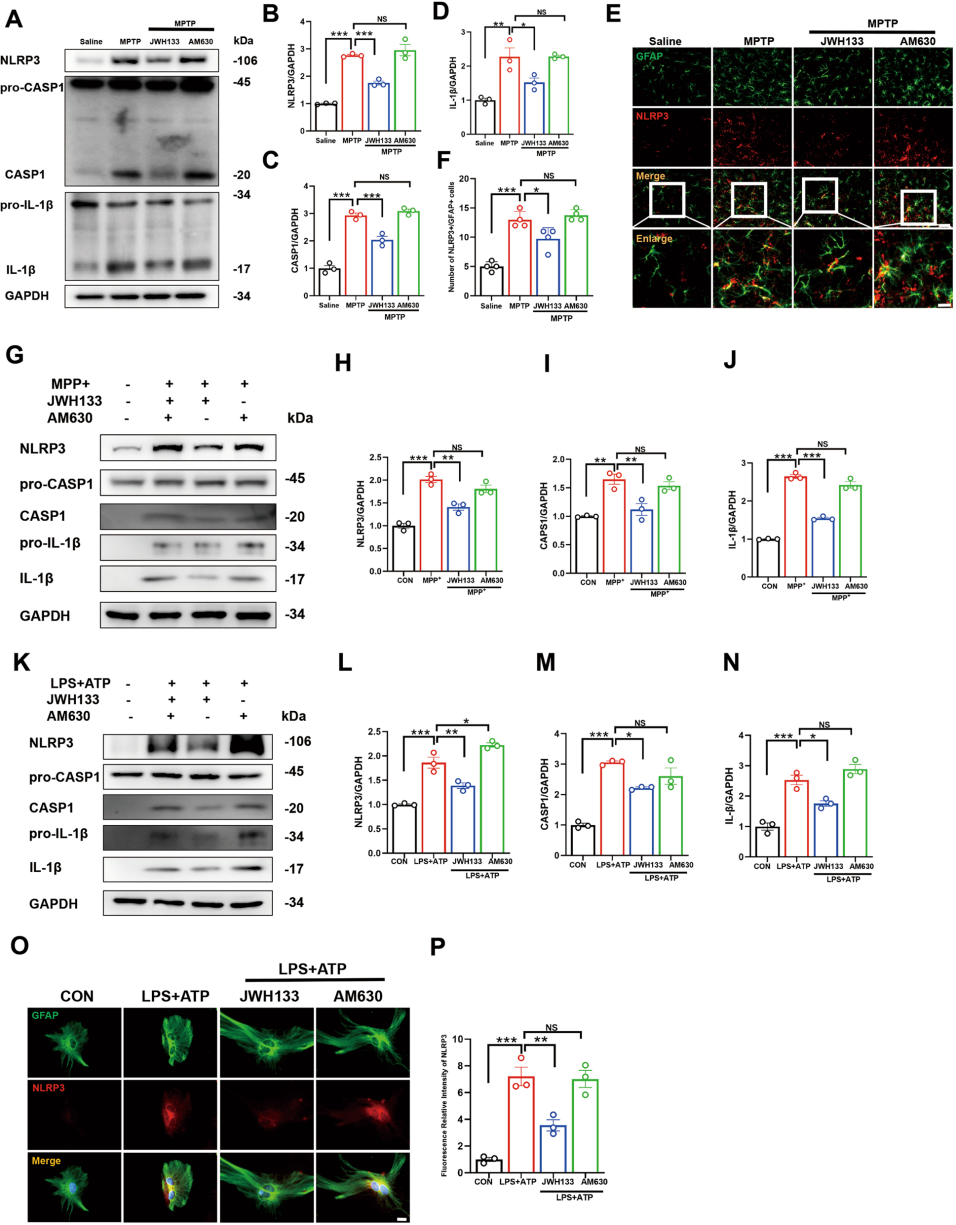
CB2R activation mitigates neuroinflammation in PD through inhibition of the NLRP3/Caspase-1/IL-1β Pathway. **A** Detection of protein levels for NLRP3, Caspase-1, pro-Caspase-1, IL-1β, and pro-IL-1β in the midbrain tissue by Western blot, with quantification shown in panels **B**, **C**, and **D** (*n* = 3). **E** Immunofluorescence staining for GFAP (green) and NLRP3 (red) in midbrain tissue of mice, with quantification shown in panel **F** (*n* = 4). Cells were treated with JWH133 (1 μM) or AM630 (1 μM) for one hour prior to stimulation with 100 μM MPP^+^ for 24 h. **G** Detection of protein levels for NLRP3, pro-Caspase-1, Caspase-1, IL-1β, and pro-IL-1β in the cells by western blot, with quantification shown in panels **H**–**J** (for 3 independent experiments). Cells were treated with JWH133 or AM630 for 1 h prior to incubation with 100 ng/mL LPS for 6 h, and then stimulated with ATP for 30 min before harvesting cell protein. **K** Detection of protein levels for NLRP3, pro-Caspase-1, Caspase-1, IL-1β, and pro-IL-1β in the cells by western blot, with quantification shown in panels **L**–**N** (for 3 independent experiments). **O** Immunofluorescence staining for GFAP (green) and NLRP3 (red) in astrocytes and the fluorescence intensity statistics of NLRP3 in **P** (for 3 independent experiments). NS means not significant, ^*^P < 0.05, ^**^P < 0.01, ^***^P < 0.001 compared with the corresponding group, as determined by the one-way ANOVA

Persistent low-grade inflammation in the brain has been reported to exacerbate neurodegenerative pathologies, including PD and Alzheimer diseases, with pro-inflammatory cytokines such as IL-1β and IL-6 considered key drivers [29–33]. Elevated levels of IL-1β, TNF-α and IL-6 have been observed in the peripheral blood of PD patients [34, 35], highlighting the importance of targeting neuroinflammation to improve PD pathology. Previous studies have suggested that CB2R may serve as potential targets for anti-inflammatory therapy [21, 22]. To investigate the modulation of neuroinflammation by CB2R in PD, we examined inflammatory factor expression in the midbrain region of mice. Q-PCR and ELISA data showed a reduction in IL-1β expression upon CB2R activation, with non-significant trends towards improvement in TNF-α and IL-6 levels (Additional file 1: Fig. S1a–f). In contrast, the CB2R antagonist did not exhibit any inhibitory effect on inflammation. Given the downregulation of IL-1β levels, we hypothesized that effects of CB2R involve pattern recognition receptors responding to endogenous stimuli and inducing aseptic inflammation. NLRs, including NLR family pyrin domain containing 1 (NLRP1), NLR family pyrin domain containing 2 (NLRP2), NLRP3, and NLR family CARD domain containing 4 (NLRC4), play a crucial role in this process within the CNS [36, 37]. To confirm the involvement of inflammasomes in CB2R-mediated anti-inflammatory effects, we assessed protein expression in the subacute model. Notably, NLRP1 and NLRP3 proteins significantly increased under MPTP treatment, while only NLRP3 protein was inhibited by the CB2R agonist JWH133 (Additional file 1: Fig. S1g–k). Subsequently, we evaluated the NLRP3-mediated pathway, NLRP3/Caspase-1/IL-1β, and found that CB2R activation inhibited this pathway (Fig. 3A–D). Immunofluorescence analysis demonstrated a reduction in NLRP3 expression on astrocytes in the midbrain following CB2R activation (Fig. 3E, F). To further investigate the relationship between CB2R on astrocytes and NLRP3-mediated neuroinflammation, we isolated and cultured primary astrocytes. Consistent with ex vivo results, cellular analysis revealed that MPP^+^ induced activation of the NLRP3/Caspase-1/IL-1β pathway, which was attenuated by CB2R agonist treatment (Fig. 3G–J). NLRP3 assembly and activation occur in response to the first signal triggered by LPS, followed by ATP stimulation as the second signal for NLRP3 inflammasome formation. Similarly, primary astrocytes stimulated with LPS and ATP demonstrated that CB2R activation reduced the release of inflammatory factors induced by this stimulation (Fig. 3K–N). Immunofluorescence analysis of astrocytes confirmed decreased NLRP3 protein expression, while CB2R inhibition did not yield the same results (Fig. 3O, P). Overall, our findings indicated that CB2R could disrupt NLRP3 inflammasome assembly, leading to the inhibition of caspase-1 activation and IL-1β maturation in vitro and in vivo.

### A key mechanism for CB2R-induced degradation of NLRP3 protein via autophagolysosomal pathway

**Fig. 4:**
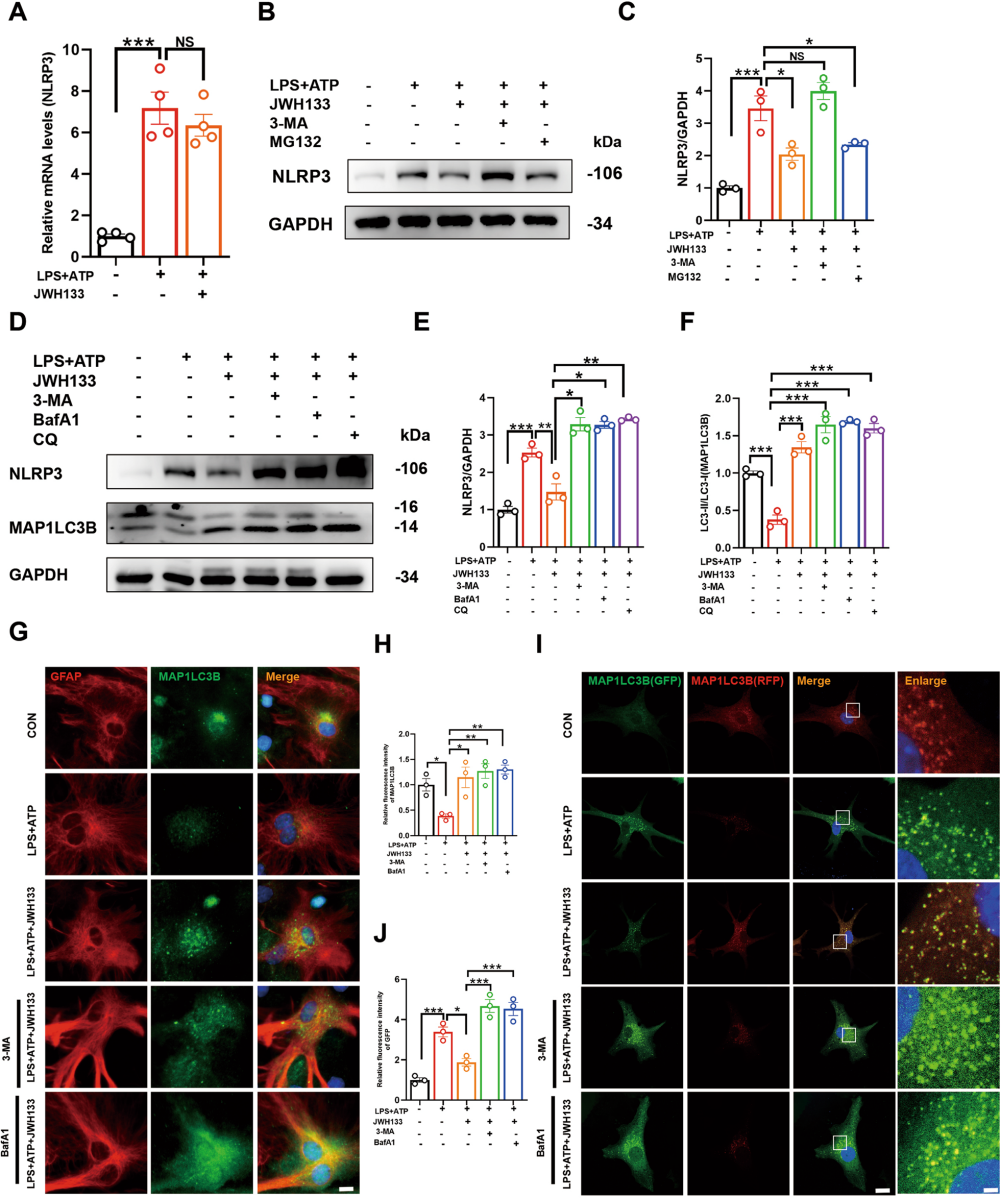
A key mechanism for CB2R-induced degradation of NLRP3 protein via autophagolysosomal pathway. **A** validation of NLRP3 mRNA levels in cells by Q-PCR (for 4 independent experiments). Primary astrocytes were pre-treated with 3-MA(5 mM) or MG132 (10 μM) for 2 h, followed by treatment with JWH133 for 1 h, and then stimulated with LPS (100 ng/mL, 6 h) and ATP (5 mM, 30 min). Protein levels of NLRP3 in cells were detected by Western blotting (**B**, **C**, for 3 independent experiments). Primary astrocytes were pre-treated with 3-MA, BafA1 (100 nM), or CQ (10 μM) for 2 h, followed by treatment with JWH133 for 1 h, and then stimulated with LPS (100 ng/mL, 6 h) and ATP (5 mM, 30 min). Expression of NLRP3 and MAP1LC3B proteins in cells was verified by western blotting (**D**), and quantified in **E**, **F** (for 3 independent experiments). **G** Primary astrocytes were treated in the same manner as described above, and immunofluorescence staining was used to analyze GFAP and MAP1LC3B in cells. Fluorescence analysis was quantified and shown in **H** (for 3 independent experiments). **I** Primary astrocytes were transfected with RFP-GFP-MAP1LC3B adenovirus for approximately 72 h, and then treated with drugs before observation under a confocal microscope. Fluorescence quantification analysis is shown in **J** (for 3 independent experiments). NS means not significant, ^*^P < 0.05, ^**^P < 0.01, ^***^P < 0.001 compared with the corresponding group, as determined by the one-way ANOVA

In this study, we aimed to elucidate the mechanism by which CB2R activation inhibits NLRP3 inflammasome activation. We investigated the effect of CB2R activation on NLRP3 expression at the mRNA transcription level and observed no significant changes (Fig. 4A), suggesting post-transcriptional regulation. Based on these findings, we hypothesized that CB2R activation may promote the degradation of NLRP3 protein. Notably, autophagy and proteasome pathways have been implicated in NLRP3 protein degradation (Fig. 4B, C). To explore the involvement of autophagic degradation, we employed the autophagy inhibitor 3-methyladenine (3-MA) and the proteasome inhibitor MG132. Intriguingly, we found that CB2R agonist JWH133 failed to degrade NLRP3 in the presence of 3-MA, indicating the dominant role of autophagy in NLRP3 degradation. To further investigate the autophagic degradation mechanism, we assessed the impact of CB2R activation on the expression of the autophagy-related protein MAP1LC3B. We observed a reduction in MAP1LC3B expression induced by LPS and ATP, and noted that the accumulation of NLRP3 could not be degraded via the autophagy–lysosome pathway. Conversely, CB2R activation accelerated NLRP3 protein degradation, which was inhibited by the autophagy inhibitor 3-MA, as well as lysosome inhibitors bafilomycin A1 and chloroquine. Immunostaining of primary astrocytes confirmed that CB2R activation promoted MAP1LC3B protein expression (Fig. 4D–F). However, inhibition of the autophagy–lysosome pathway also impaired MAP1LC3B protein degradation (Fig. 4G, H). To assess autophagic flux, we utilized a tandem fluorescence mRFP-GFP-MAP1LC3B adenovirus to transduce primary astrocytes. GFP fluorescence is sensitive to the acidic environment within lysosomes, while RFP remains relatively stable. Therefore, the merging of GFP and RFP fluorescence indicates fusion between autophagosomes and lysosomes, while exclusive green GFP fluorescence denotes a blockade in autophagic flux. Our results revealed increased green fluorescence intensity in cells treated with LPS + ATP, while CB2R activation enhanced GFP-RFP fusion, indicative of augmented autophagy, which was impeded by autophagy–lysosome pathway inhibitors (Fig. 4J–I). Collectively, our findings suggested that CB2R activation promoted the initiation of autophagic flux, thus facilitating NLRP3 protein degradation.

### Foxg1 is a crucial regulatory molecule for CB2R-mediated autophagy promotion

**Fig. 5:**
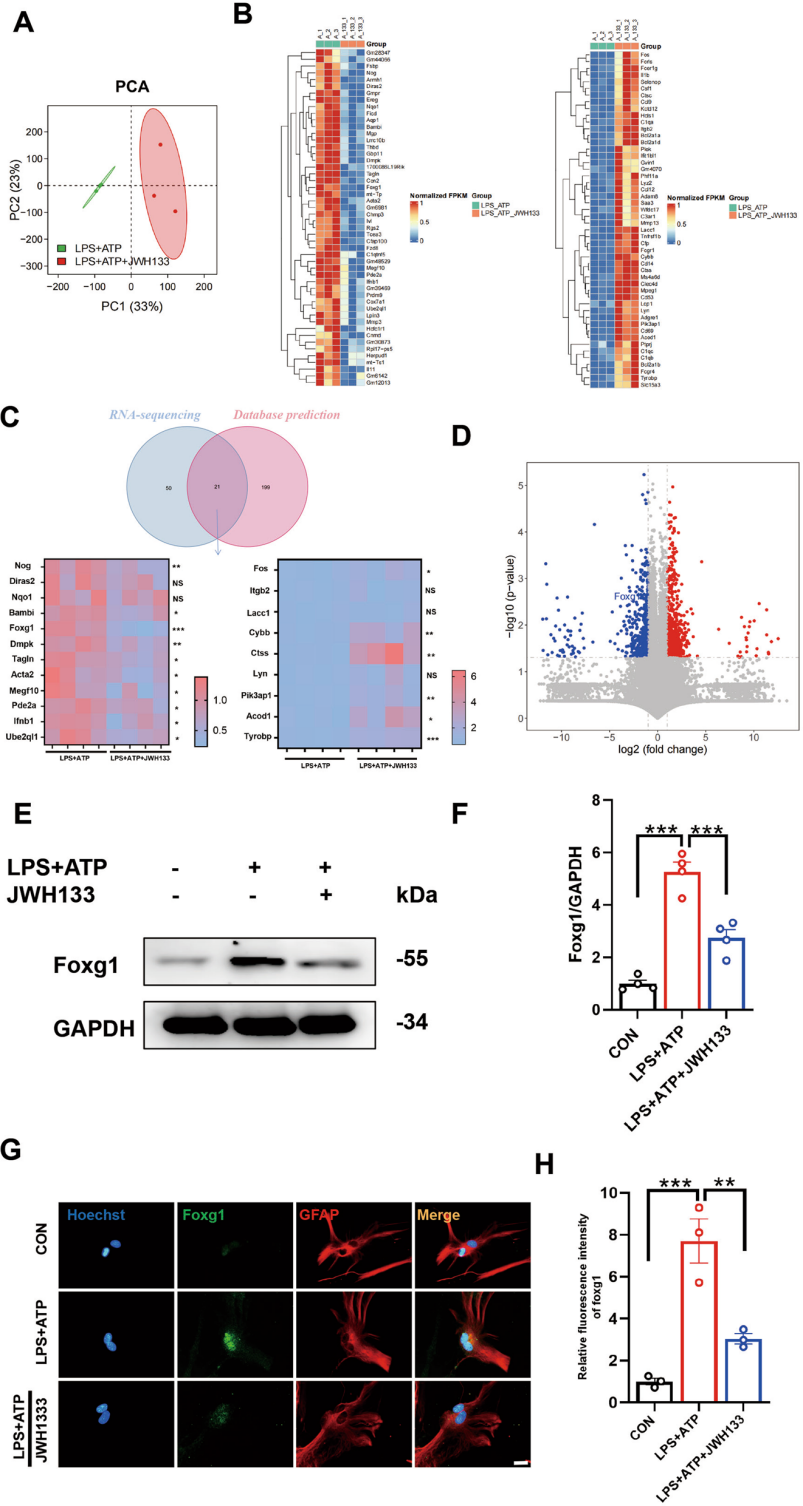
Foxg1 is a crucial regulatory molecule for CB2R-mediated autophagy promotion. **A** Principal Component Analysis (PCA) plot depicting the transcriptomic profiles of (specify the samples or conditions being compared). Each data point represents an individual sample, and the plot reveals the multidimensional relationships between samples based on their gene expression patterns. **B** Differential genes between LPS + ATP vs LPS + ATP + JWH133 groups (downregulated genes on the left, upregulated genes on the right). **C** The study involves retrieving the intersection between genes in a database and genes associated with sequencing changes. Subsequently, approximately 20 genes from this intersection will be selected for qPCR analysis. **D** Location of foxg1 among differentially expressed genes in volcano plot. **E** Western blot analysis was performed to assess the expression of foxg1. The corresponding representative blot is presented in **F** (for 4 independent experiments.). **G** Immunofluorescent staining of primary astrocytes (foxg1 is green, GFAP is red), and the fluorescence intensity statistics of foxg1 are shown in **H** (for 3 independent experiments). NS means not significant, ^*^P < 0.05, ^**^P < 0.01, ^***^P < 0.001 compared with the corresponding group, as determined by the one-way ANOVA

**Fig. 6:**
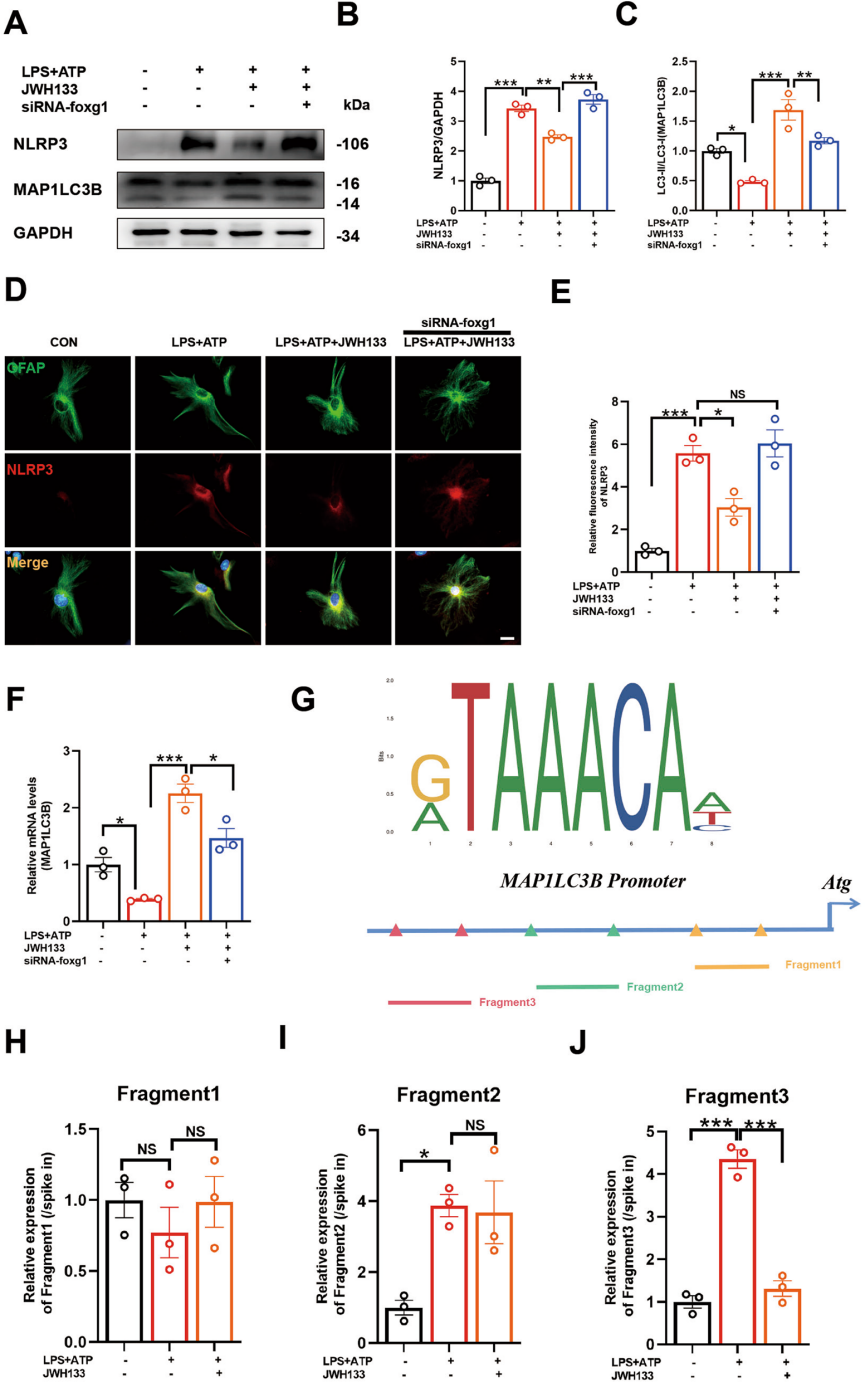
Foxg1 is a crucial regulatory molecule for CB2R-mediated autophagy promotion. **A** Protein detection of NLRP3 and MAP1LC3B in primary astrocytes. Statistical data for the proteins are presented in **B**, **C,** respectively (for 3 independent experiments). **D** Immunofluorescence staining of primary astrocytes showing GFAP (green fluorescence) marking astrocytes and NLRP3 protein (red fluorescence) detection. Fluorescence intensity data for NLRP3 are shown in **E** (for 3 independent experiments). **F** Expression levels of MAP1LC3B mRNA detected using Q-PCR (For 3 independent experiments). **G** Potential binding sites of foxg1 on the promoter region of MAP1LC3B. Three putative binding sites of foxg1 on the promoter region of MAP1LC3B were identified using cut&run assay (**H**–**J**, for 3 independent experiments). NS means not significant, ^*^P < 0.05, ^**^P < 0.01, ^***^P < 0.001 compared with the corresponding group, as determined by the one-way ANOVA

To explore the underlying mechanisms of CB2R regulation of autophagy, we conducted transcriptomic sequencing on primary astrocytes after CB2R activation (Fig. 5A). We compared about the top 50 upregulated and downregulated genes in astrocytes with CB2R activation to the LPS and ATP group (Fig. 5B). We also searched the GeneCards database (https://www.genecards.org/) and relevant literature for 220 genes associated with autophagy–lysosome. By intersecting these genes with the differentially expressed genes from sequencing, we identified 21 common genes. Subsequent PCR testing confirmed significant differences in genes such as nucleolar gTP-binding protein 1 (Nog1), dystrophia myotonica protein kinase (Dmpk), cytochrome b-245 beta chain (Cybb), cathepsin S (CTSS) consistent with the sequencing results (Fig. 5C). However, the most significant changes were observed in foxg1 and TYRO protein tyrosine kinase binding protein (Tyrobp). Literature review revealed that foxg1 is involved in autophagy and potentially interacts with the MAP1LC3B protein [38, 39] (Fig. 5D). Thus, we hypothesized that foxg1 might be a potential target for CB2R-mediated regulation of autophagy. Western blot results supported this hypothesis, showing increased foxg1 protein levels with LPS and ATP treatment and reduced production after CB2R activation (Fig. 5E, F). Immunofluorescence staining of primary astrocytes demonstrated that foxg1 is primarily expressed in the nucleus as a transcription factor, and its expression significantly decreased after CB2R activation (Fig. 5G, H). Considering the properties of the foxg1 protein, we speculated whether foxg1 acts as a transcription factor influencing MAP1LC3B expression. We interfered with foxg1, abolishing the CB2R-mediated promotion of autophagy and the degradation of NLRP3, leading to a decrease in MAP1LC3B protein levels (Fig. 6A–C). Immunostaining results for NLRP3 in primary astrocytes supported this finding (Fig. 6D, E). Therefore, we proposed that foxg1 might function as a transcription factor, regulating MAP1LC3B expression at the transcriptional level.

To validate this hypothesis, PCR data confirmed that JWH133 promoted MAP1LC3B mRNA levels (Fig. 6F). However, interfering with foxg1 resulted in decreased MAP1LC3B mRNA levels. Prediction using NCBI (https://www.ncbi.nlm.nih.gov/), JASPAR (https://jaspar.elixir.no/) and UniProt databases (https://www.uniprot.org/) indicated a potential binding site (5’-GTAAACAA-3’) between foxg1 and MAP1LC3B. Three potential binding fragments in the promoter region of MAP1LC3B were predicted (Fig. 6G) (fragment1: − 82 bp to 331 bp; − 566 bp to 810 bp; − 1410 bp to 1650 bp), and analysis using CUT&RUN technique suggested that fragment 3 might be the potential site where CB2R influences the interaction between foxg1 and MAP1LC3B(Fig. 6H–J).

Collectively, the aforementioned evidence demonstrated that foxg1 is an important molecule influencing the transcription of MAP1LC3B. CB2R activation inhibited the interaction between foxg1 and MAP1LC3B, thereby promoting MAP1LC3B transcription.

### Knockdown of foxg1 exacerbates MPTP-induced PD-like symptoms in mice

**Fig. 7:**
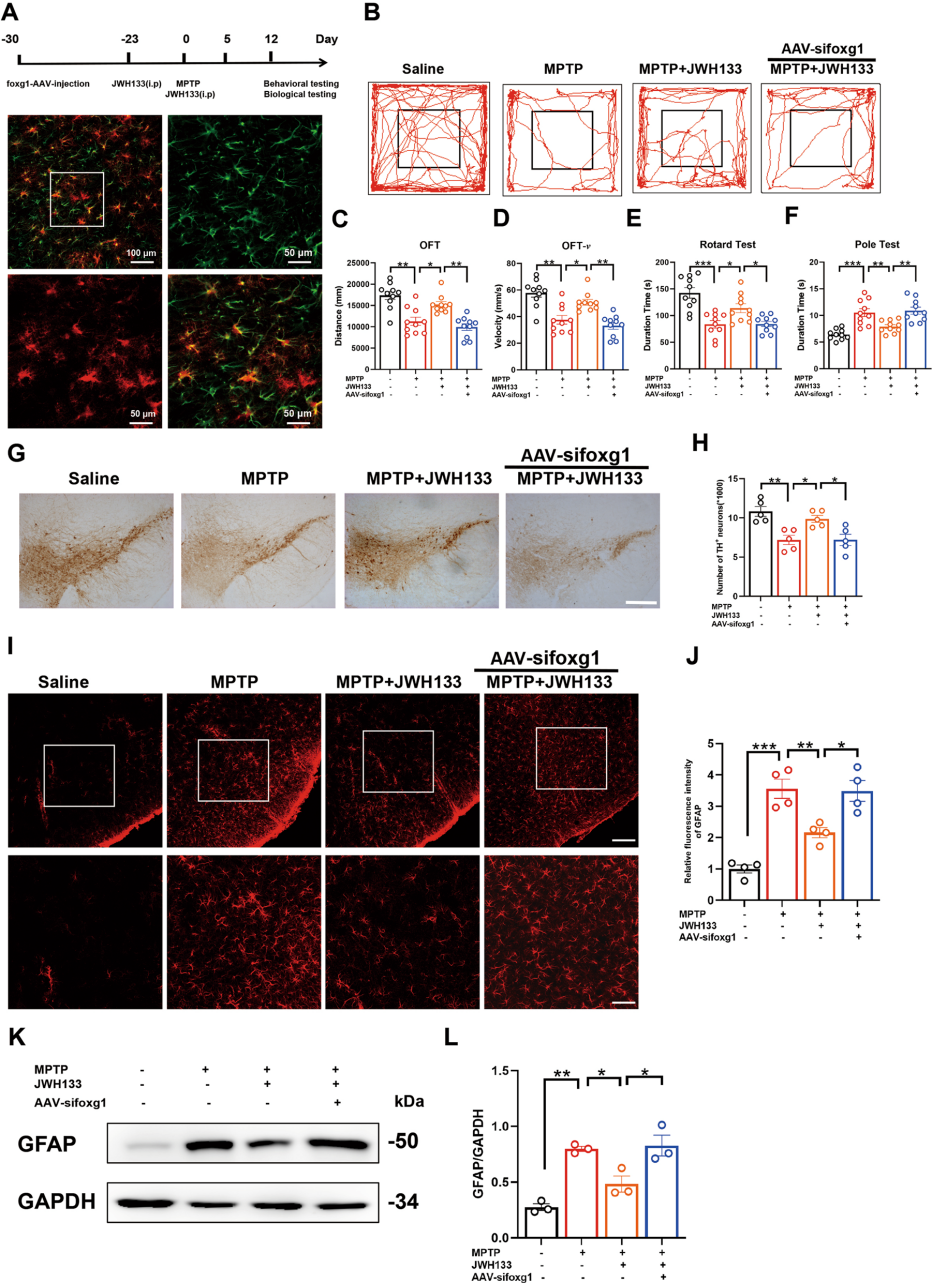
Knockdown of foxg1 exacerbates MPTP-induced PD-like symptoms in mice. **A** Experimental flow chart. Mice were injected with foxg1 AAVs into the midbrain 30 days prior to the experiment (green indicates midbrain astrocytes marked with GFAP, and red indicates the injected adenovirus, with a scale bar of 50 μm). Approximately 23 days later, mice were intraperitoneally injected with CB2R agonist JWH133 for 7 consecutive days. Subsequently, mice were subcutaneously injected with MPTP for 5 consecutive days, and JWH133 was also injected during these 5 days. Behavioral and biological tests of mice were performed thereafter. **B** The trajectory of the mice in the open field test, with distance traveled and time shown in **C** and **D** (*n* = 10), respectively. **E**, **F** Time recorded for each mouse in the rotarod test and pole test, respectively (*n* = 10). **G** Immunohistochemistry staining for TH-positive neurons in the midbrain of mice (scale bar, 200 μm), with stereological counts shown in **H** (*n* = 5). **I** Immunofluorescence detection of activated astrocytes in the midbrain of mice using GFAP as a marker (scale bar, 200 μm in the top panel, 100 μm in the bottom panel), with fluorescence quantification shown in **J** (*n* = 4). **K**, **L** Western blot detection of TH expression in midbrain and corresponding statistical data (*n* = 3). NS means not significant, ^*^P < 0.05, ^**^P < 0.01, ^***^P < 0.001 compared with the corresponding group, as determined by the one-way ANOVA

In vitro studies on primary astrocytes have demonstrated that knocking down foxg1 abolished the effects of JWH133, resulting in suppression of autophagy and failure in NLRP3 degradation. However, to investigate the impact of foxg1 on PD-like manifestations in an in vivo animal model, we targeted the SNc region of mice with a specific viral vector for foxg1 knockdown in astrocytes. After 23 days, the mice were treated with the CB2R agonist JWH133 while establishing a subacute model of MPTP-induced PD (Fig. 7A). Behavioral data revealed that knockdown of foxg1 abrogated the beneficial effects of JWH133 on motor behavior in mice. Specifically, mice with foxg1 knockdown exhibited reduced distance covered and slower movement speed in the open field test (Fig. 7B–D), decreased time spent on the rotarod test (Fig. 7E), and increased time required to climb the rod in the pole test (Fig. 7F). The number of TH-positive cells in the midbrain also decreased (Fig. 7G, H), accompanied by activation of astrocytes in the midbrain (Fig. 7I–L). Therefore, these findings suggested that in an in vivo PD model, knockdown of foxg1 in astrocytes negated the effects of JWH133, leading to worsened PD-like pathological features in mice.

### Knockdown of foxg1 hampers autophagic response in astrocytes and inhibits NLRP3 degradation

**Fig. 8:**
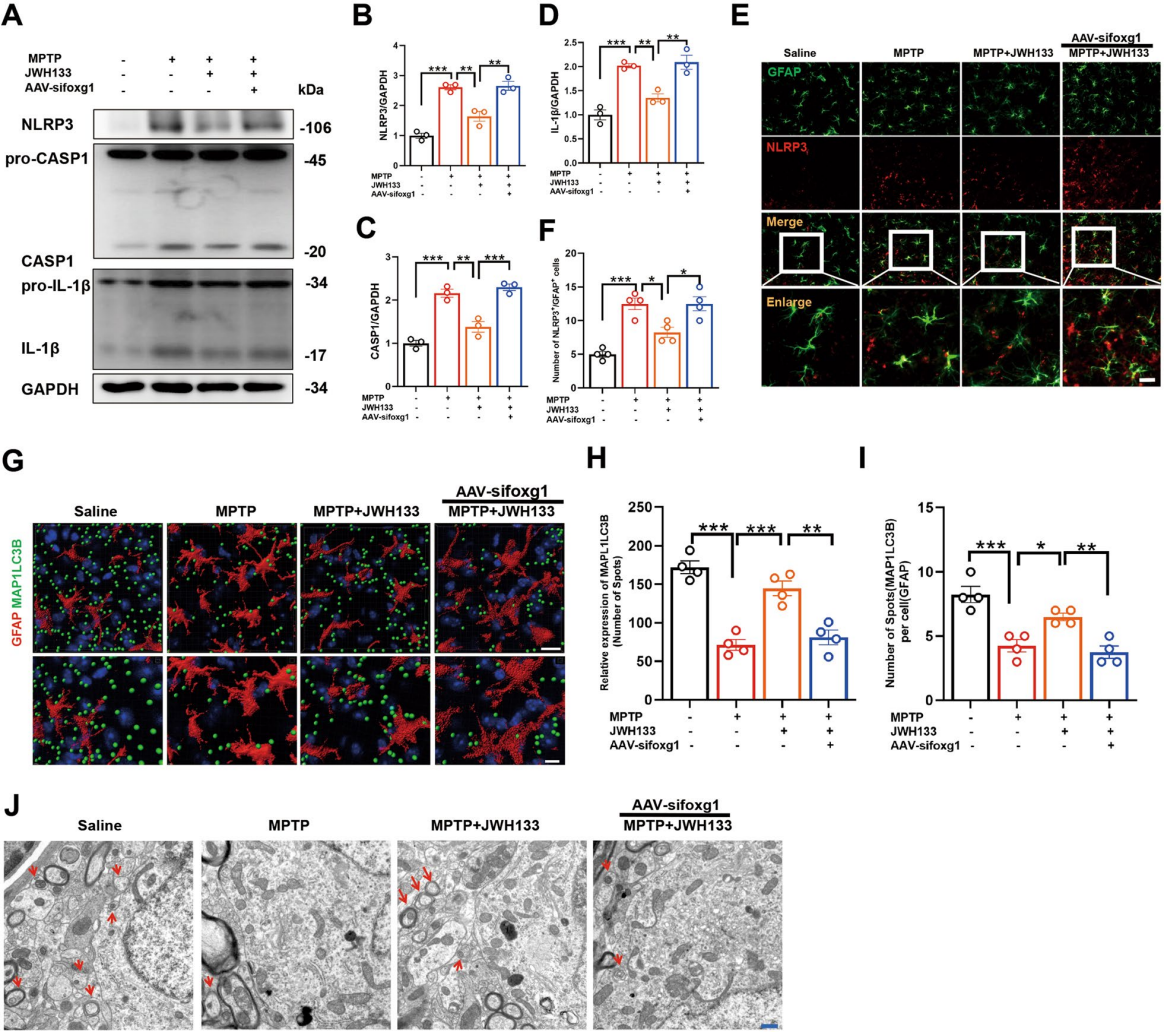
Knockdown of foxg1 hampers autophagic response in astrocytes and inhibits NLRP3 degradation. **A** Detection of protein levels for NLRP3, Caspase1, pro-Caspase1, IL-1β, and pro-IL-1β in the midbrain tissue by western blot, with quantification shown in panels **B**–**D** (*n* = 4). **E** Immunofluorescence staining for GFAP (green) and NLRP3 (red) in midbrain tissue of mice, with quantification shown in panel **F**–**H** (*n* = 4). **G** Mouse midbrain GFAP (red) and MAP1LC3B (green) immunofluorescence staining, the captured image was processed by imaris software, and the fluorescence statistics are shown in **H**, **I** (*n* = 4). **J** Autophagosomes (marked by red arrows) observed by transmission electron microscope in mouse midbrain tissue. NS means not significant, ^*^P < 0.05, ^**^P < 0.01, ^***^P < 0.001 compared with the corresponding group, as determined by the one-way ANOVA

Subsequently, we examined the NLRP3/Caspase-1/IL-1β pathway in the midbrain tissue of mice and found that JWH133 suppressed the activation of this pathway. However, upon knockdown of foxg1, the effect of JWH133 was nullified (Fig. 8A–D), leading to increased protein accumulation of NLRP3 in astrocytes and exacerbating neuroinflammation (Fig. 8E, F). Moreover, the expression of MAP1LC3B, a marker for autophagy, was reduced in astrocytes of the midbrain (Fig. 8G–I). Transmission electron microscopy observation of midbrain autophagosomes confirmed that knockdown of foxg1 inhibited the formation of autophagosomes (Fig. 8J). Therefore, these results indicated that foxg1 is a crucial molecule for CB2R-mediated promotion of autophagic response and acceleration of NLRP3 degradation.

## Discussion

Our present study provided important insights into the intricate relationship between CB2R and NLRP3 in the context of PD. Our findings shed light on the underlying mechanisms through which CB2R activation regulates inflammatory pathways and autophagic response in astrocytes, thereby influencing PD pathology. One significant aspect of our study is investigating the modulation of neuroinflammation through the activation of CB2R. Previous research has suggested that neuroinflammation plays a crucial role in the pathogenesis of PD [3, 37]. In our study, we focused on PD mice and detected notable activation of NLRP1 and NLRP3 inflammasomes in their midbrain tissue. However, when we activated CB2R using JWH133, we observed a significant suppression of the NLRP3 inflammasome pathway, leading to reduced expression of IL-1β. Our analysis primarily concentrated on cytokine and inflammasome component expression at the tissue level. To further explore the mechanism, we conducted cell experiments in vitro and found that CB2R activation also attenuated the activation of the NLRP3/Caspase-1/IL-1β pathway induced by MPP + or LPS and ATP on astrocytes. Our findings are in line with previous reports that have shown CB2R activation to be beneficial in other CNS-related diseases, such as multiple sclerosis and stroke. For instance, in experimental models of demyelinating, neurometabolic disease, CB2R activation effectively inhibited axonal damage through the normalization of altered redox and lipid homeostatic pathways [40]. In stroke models, CB2R activation has exhibited neuroprotective effects by reducing infarct size and inhibiting the inflammatory response [25]. Considering the importance of inhibiting NLRP3 protein synthesis or enhancing its degradation and metabolism to ameliorate PD-like pathological changes, our study uncovered that CB2R does not directly regulate NLRP3 synthesis. Instead, it facilitates the degradation of NLRP3 within the brain. This degradation process is accelerated through the autophagy–lysosome pathway, rather than the proteasome pathway.

Autophagy, a crucial cellular process involved in recycling and eliminating damaged cellular components, is essential for maintaining cellular homeostasis [41, 42]. When autophagy in astrocytes is impaired, there is an accumulation of aberrant protein aggregates and dysfunctional organelles [42, 43]. Consequently, astrocytes become less efficient in supporting neuronal health and exhibit reduced antioxidant defenses, leading to increased neuroinflammation. In mouse models of PD, the dysregulated autophagy in astrocytes has been associated with enhanced neurodegeneration, thereby exacerbating the progression of PD pathology. The compromised support from astrocytes further contributes to the loss of dopaminergic neurons and worsens motor deficits in PD mice. In our research, we confirmed that activation of CB2R promotes autophagic response in astrocytes, leading to an increase in the expression of MAP1LC3B, a crucial molecule involved in autophagosome formation. In an in vivo model, the expression of MAP1LC3B was downregulated in response to MPTP induction, but CB2R activation was found to enhance the expression of MAP1LC3B in astrocytes, while also accelerating the degradation of NLRP3 protein. This resulted in reduced loss of TH-positive neurons in the midbrain of mice.

**Fig. 9:**
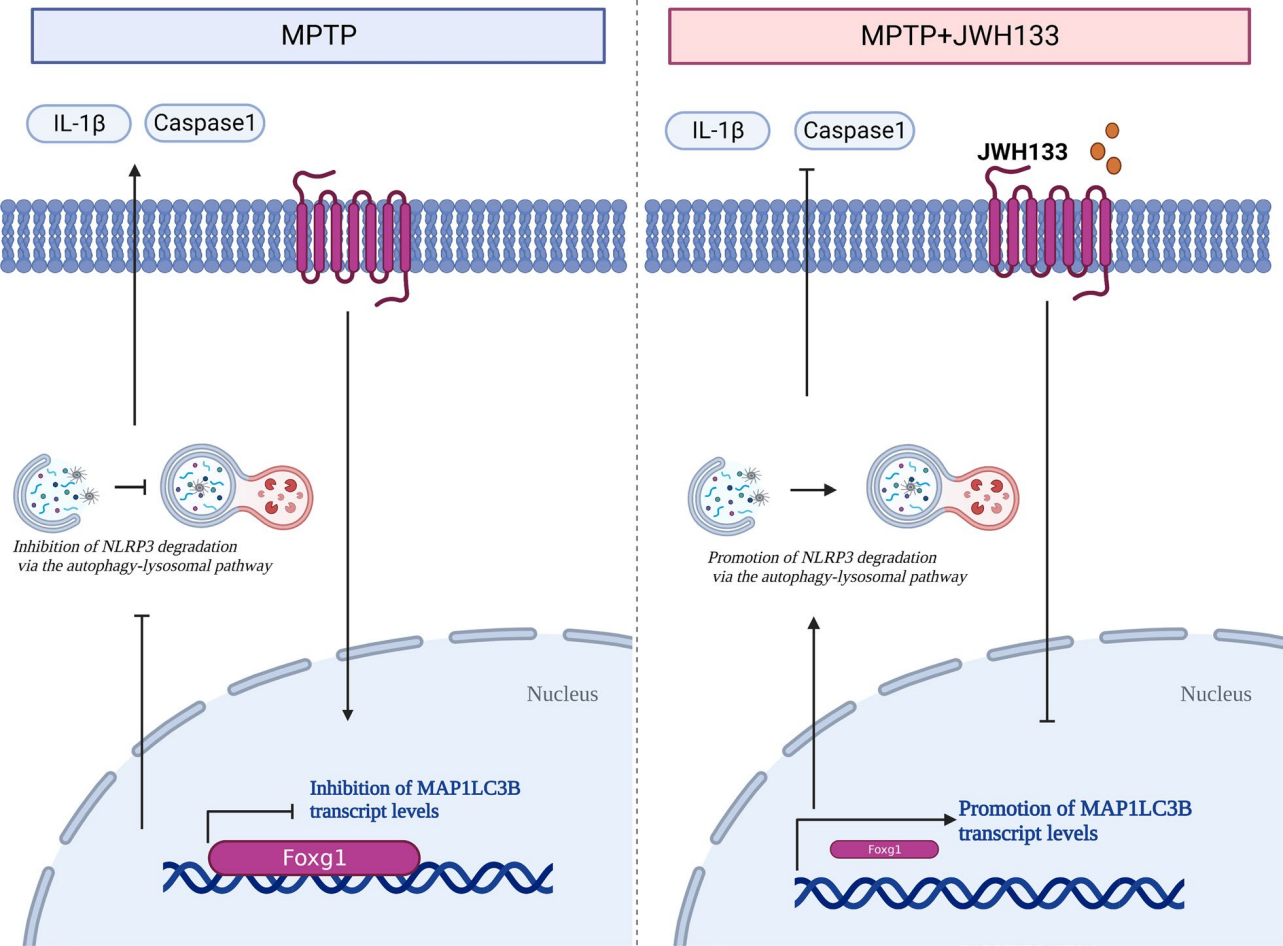
Targeting CB2R in astrocytes for Parkinson's disease therapy: unraveling the foxg1-mediated neuroprotective mechanism through autophagy-mediated NLRP3 degradation. activation of CB2R reduced the binding of foxg1 to the promoter region of MAP1LC3B, thereby promoting the transcription of MAP1LC3B and increasing the autophagic level in astrocytes. This led to an enhanced autophagic degradation of NLRP3 and a reduction in the release of caspase-1 and IL-1β. As a result, neurodegeneration was alleviated in PD mice. These findings suggest that CB2R activation plays a crucial role in regulating autophagy and may serve as a potential therapeutic target for neurodegenerative disorders

Subsequently, we focused on understanding the impact of CB2R activation on autophagic response. By performing transcriptomic sequencing, we compared the changes in autophagy-related genes under inflammatory stimulation induced by LPS and ATP. Notably, we observed significant alterations in genes including foxg1, Dmpk, Cybb, and Tyrobp. Through validation experiments, we identified foxg1 as the pivotal regulator of autophagy. Following LPS and ATP stimulation, primary astrocytes showed increased autophagy levels upon CB2R activation, which was accompanied by the downregulation of foxg1 expression. Previous studies have suggested that foxg1 played a critical role in the auditory degeneration process by regulating macroautophagy/autophagy and influencing the sensitivity of aging hair cells to inflammation through autophagy pathways [44, 45]. However, our research revealed distinct mechanisms through which foxg1 modulates autophagy in astrocytes. We found that foxg1 acted as a transcriptional regulator of MAP1LC3B and identified approximate binding sites of foxg1 on the MAP1LC3B promoter, a novel discovery not reported in previous studies. Furthermore, in vitro experiments involving foxg1 interference in primary astrocytes demonstrated the abrogation of CB2R agonist effects, resulting in decreased cellular autophagy levels and impaired NLRP3 degradation. Our in vivo experiments utilizing a subacute model of MPTP-induced PD provided additional evidence supporting the crucial role of foxg1 in CB2R-mediated neuroprotection. Knockdown of foxg1 in astrocytes within the SNc region led to worsened motor impairments, decreased TH-positive cell count, and enhanced astrocyte activation. These findings underscore the significance of foxg1 in maintaining dopaminergic neuronal integrity and regulating astrocytic responses in the context of PD. However, it is important to acknowledge that the selective knockdown of foxg1 in astrocytes may have indirect effects on other cell types within the SNc, and future studies employing cell-specific knockout or conditional knockout approaches would provide a more precise understanding of the role of foxg1 in PD pathogenesis (Fig. 9).

Our research provided a novel perspective on the association between CB2R in astrocytes and neuroinflammation in PD. Specifically, CB2R activation stimulated the autophagy–lysosome pathway, leading to accelerated degradation of the NLRP3 protein. In this process, foxg1 played a crucial role, as CB2R activation downregulated foxg1 expression, thereby reducing its binding to the MAP1LC3B promoter region (− 1410 to − 1650 bp) and promoting MAP1LC3B generation, which in turn facilitated NLRP3 degradation. In summary, CB2R emerged as a promising therapeutic target for CNS-related diseases, owing to its anti-inflammatory and neuroprotective effects. The ability of CB2R to suppress NLRP3 expression in glial cells offers a potential mechanism to alleviate neuroinflammation, a common feature of CNS disorders. As research on CB2R advances, harnessing its immunomodulatory potential may hold significant promise in developing innovative therapeutic strategies for various CNS-related diseases. Nonetheless, our study exhibits multidirectional limitations, such as its general applicability to other central nervous system diseases. Although our research indicates that activation of the CB2R might be beneficial for other CNS disorders, this assumption is largely conjectural. Furthermore, the study primarily concentrates on the role of astrocytes in PD. While astrocytes are essential in neuroinflammation and the pathology of PD, other cell types, such as neurons, microglia, and oligodendrocytes, also play vital roles in the pathophysiology of the disease. A more comprehensive methodology that encompasses these additional cell types could offer a more thorough understanding of the disease. Lastly, the translation of these findings into clinical applications represents a significant challenge. The journey from preclinical research to human trials is complex and necessitates meticulous consideration of dosage, administration, and patient selection. Thus, there remains a lengthy path to be explored.

### Supplementary Information

**Additional file 1: Figure S1.** CB2R activation mitigates neuroinflammation in PD through inhibition of the NLRP3/Caspase-1/IL-1β Pathway. (a-c) Detection of mRNA levels for TNF-α, IL-6, and IL-1β in the midbrain of mice by qPCR (*n* = 4), and detection of protein expression levels for TNF-α, IL-6, and IL-1β in the midbrain via ELISA (d-f, *n* = 5). (g)Protein expression levels for NLRP1, NLRP2, NLRP3, and NLRC4 in the midbrain tissue, with quantification for each marker shown in panels h–k (*n* = 3). NS means not significant, ^*^P < 0.05, ^**^P < 0.01, ^***^P < 0.001 compared with the corresponding group, as determined by the one-way ANOVA.

## Data Availability

The raw data that support the findings of this study are available from the corresponding author, upon reasonable request.

## Abbreviations

PD: Parkinson's disease
GPCR: G protein-coupled receptor
SNc: Substantia nigra pars compacta
TH: Tyrosine hydroxylase
ATP: Adenosine triphosphate
IL-1β: Interleukin-1β
TNF-α: Tumor necrosis factor-α
NLRP3: NOD-like receptor family pyrin domain containing 3
IL-18: Interleukin-18
CB2R: Cannabinoid receptor 2
CNS: Central nervous system
GFAP: Glial fibrillary acidic protein
MAP1LC3B: Microtubule-associated protein 1 light chain 3 beta
NLRP1: NLR family pyrin domain containing 1
NLRP2: NLR family pyrin domain containing 2
NLRC4: NLR family CARD domain containing 4
