# Supplementary figures and images for: Targeting CB2R in astrocytes for Parkinson's disease therapy: unraveling the Foxg1-mediated neuroprotective mechanism through autophagy-mediated NLRP3 degradation
Source: J Neuroinflammation. 2023 Dec 19;20:304. doi: 10.1186/s12974-023-02989-2 (PMC10729372; doi:10.1186/s12974-023-02989-2)

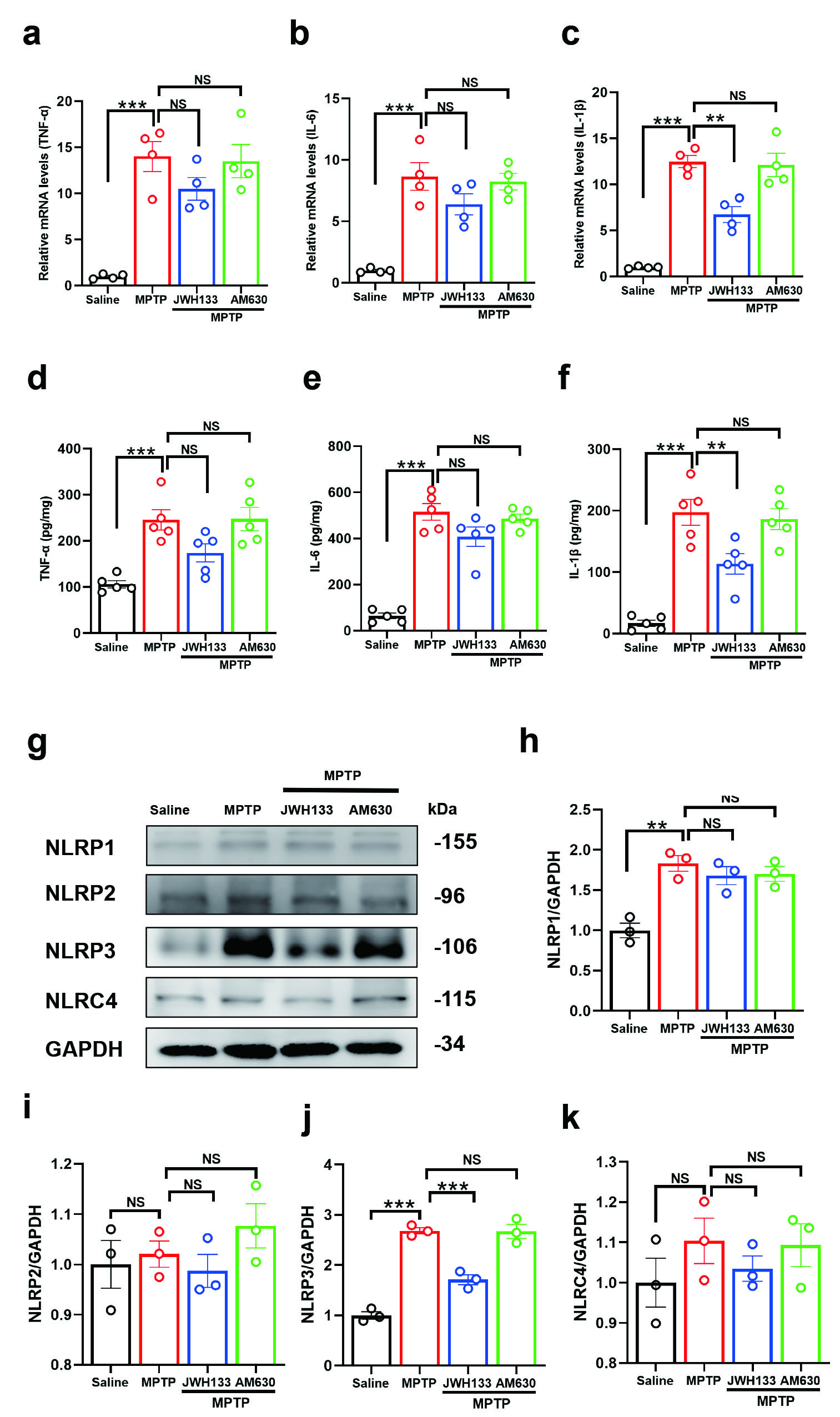

Supplement: Supplementary file 1 — Additional file 1: Figure S1. CB2R activation mitigates neuroinflammation in PD through inhibition of the NLRP3/Caspase-1/IL-1β Pathway. (a-c) Detection of mRNA levels for TNF-α, IL-6, and IL-1β in the midbrain of mice by qPCR (n = 4), and detection of protein expression levels for TNF-α, IL-6, and IL-1β in the midbrain via ELISA (d-f, n = 5). (g)Protein expression levels for NLRP1, NLRP2, NLRP3, and NLRC4 in the midbrain tissue, with quantification for each marker shown in panels h–k (n = 3). NS means not significant, *P < 0.05, **P < 0.01, ***P < 0.001 compared with the corresponding group, as determined by the one-way ANOVA. [file 12974_2023_2989_MOESM1_ESM.jpg]
